# Emergency Vaccination as a Control Strategy against Sheeppox Outbreak in a Highly Susceptible Population

**DOI:** 10.3390/ani12162084

**Published:** 2022-08-15

**Authors:** Atef Oreiby, Ayman S. Seada, Mohamed F. Abou Elazab, Walied Abdo, Mohamed Kassab, Yamen Hegazy, Hazim O. Khalifa, Tetsuya Matsumoto

**Affiliations:** 1 Department of Animal Medicine (Infectious Diseases), Faculty of Veterinary Medicine, Kafrelsheikh University, Kafr El-Sheikh 33516, Egypt; 2 Bacteriology Department, Animal Health Research Institute, Tanta Branch, Egypt; 3 Department of Clinical Pathology, Faculty of Veterinary Medicine, Kafrelsheikh University, Kafr El-Sheikh 33516, Egypt; 4 Pathology Department, Faculty of Veterinary Medicine, Kafrelsheikh University, Kafr El-Sheikh 33516, Egypt; 5 Cytology and Histology Department, Faculty of Veterinary Medicine, Kafrelsheikh University, Kafr El-Sheikh 33516, Egypt; 6 Department of Infectious Diseases, Graduate School of Medicine, International University of Health and Welfare, Narita 286-0048, Japan; 7 Department of Pharmacology, Faculty of Veterinary Medicine, Kafrelsheikh University, Kafr El-Sheikh 33516, Egypt; 8 Antimicrobial Resistance Research Center, National Institute of Infectious Diseases, Higashi Murayama, Tokyo 189-0002, Japan

**Keywords:** sheeppox, emergency vaccination, Egypt, control, outbreak

## Abstract

**Simple Summary:**

Sheeppox is an emerging disease in different parts of the world with a huge impact on animal health and production. Emergency vaccination might be an effective strategy to overcome this disease. In this study we characterized in detail an outbreak of sheeppox in a susceptible naive sheep population in Egypt. The local and the systemic signs as well as postmortem and the histopathological findings of the disease were confirmed. We also compared the efficacies of two commercial sheeppox vaccines (Romanian strain and RM-65 vaccines). Our results confirmed the superiority of the RM-65 vaccine compared to the Romanian strain vaccine in protecting the animals. Furthermore, we highlighted the validity of emergency vaccination to control a sheeppox outbreak.

**Abstract:**

This study aimed to investigate a sheeppox outbreak in a highly susceptible naive sheep population in Kharsit village, Gharbia Governorate, Egypt. Moreover, to compare commercial sheeppox vaccines, the Romanian strain and RM-65 vaccines, as emergency vaccination against sheeppox under field conditions. In December 2018, a sheeppox outbreak occurred in a flock of 65 sheep upon the purchase of an apparently healthy ewe from outside the village. This ewe showed a systemic disease with cutaneous lesions after a few days, thereafter more cases began to appear. Cutaneous lesions in other sheep in the flock in the form of macules, papules, and scabs were common in wool-less areas of the body, in addition to fever and respiratory disorders. Postmortem findings revealed the congestion of visceral organs with apparent gross pathology of the lung. Biopsies of cutaneous lesions and visceral organs were collected, and sheeppox was identified by histopathology and transmission electron microscopy, which showed the existence of sheeppox cells and intracytoplasmic brick-shape sheeppox virions. The Romanian strain and RM-65 vaccines were used for the emergency vaccination for two different groups of animals and the third group was left as a control group. Serum samples were collected before vaccination as well as 21 days post-vaccination, and serum protein fractionation analysis was performed for all groups. The outbreak ended after 2.5 months, the cumulative incidence was 66.2%, and the overall case fatality was 51.1%. There was significantly higher protection against sheeppox infection and mortalities among RM-65 vaccine immunized group compared to Romanian strain vaccine-immunized animals at *p* < 0.05. RM-65-vaccinated animals did not show sheeppox cases or mortalities, compared to Romanian strain-vaccinated animals, which had mild pox signs in 78% of animals and case fatality of 35.7%. The serum protein analysis also indicated the superior performance of the RM-65 vaccine; it increased the level of α1-globulin and β-globulin compared to the Romanian strain, which increased the level of β-globulin only. The current study shows a better performance of the tested RM-65 than the Romanian strain vaccine for emergency vaccination against sheeppox under field conditions. These findings point to the validity of emergency vaccination against sheeppox and the importance of the comparative field evaluation of vaccines; however, wide-scale studies are required for further evaluation. Future investigation of whether the Romanian strain itself or vaccine-production-related issues are responsible for these findings is required.

## 1. Introduction

Meat, wool, and leather production from sheep are greatly affected by sheeppox, which also has a negative effect on the export of sheep [1]. Rawlins et al. (2022) estimated losses from sheep- and goatpox in Nigeria to range from GBP 5 to 30 per reproductive female under different production systems and severity of illness [2]. Sheeppox virus (SPV) survives for long periods in the environment, and the transmission of infection occurs through inhalation and contact [3]. The disease is distributed in Africa, the Middle East, Asia, Turkey, Bulgaria, and Greece [4].

Immunity against Capripox viruses can provide protection against the disease [4]. Accordingly, the clinical outcome of sheeppox virus infection will be no disease, cutaneous pox lesions, and/or visceral involvement, according to the animal’s immunity, where highly susceptible animals will develop the fatal severe disease [5]. Sheeppox virus is antigenically related to other Capripox viruses, vaccination is considered the cornerstone to control this disease, as the restriction of animals’ movement and quarantine are not enough to control Capripox outbreaks [4]. Highly immunized animals will not show symptoms of the disease, in contrast to partially immune or immune-susceptible animals because severity is affected by immune status [1].

Vaccines against Capripox viruses are usually live attenuated, which stimulate cellular as well as humoral immune responses. Romanian, RM-65, and Kenyan live-attenuated sheeppox strains vaccines are commonly used to protect sheep against sheeppox [6]. Protective antibodies following vaccination take a relatively long time to manifest; in a previous study of goatpox vaccine-immunized sheep, the authors began to collect serum samples for the detection of antibodies 40 days post-vaccination [7]. Similarly, in a previous study to protect against lumpy skin disease in cattle using the Romanian strain sheeppox vaccine, antibodies appeared after 7 days and reached a protective level 21 days post-vaccination [8]. Therefore, post-vaccinal antibody production does not facilitate the emergency use of Capripox vaccines. Although humoral immunity is not without value and may provide protection, the protection against Capripox viruses is mainly dependent on cell-mediated immunity [9]. Cell-mediated immunity enables the emergency vaccination against Capripox viruses, as had been frequently reported against the lumpy skin disease of cattle [8,10] but it had not been reported clearly in previous studies regarding sheeppox.

In Egypt, the disease is endemic [11]; however, its epidemiological features are not identified. There are commercially available, live attenuated sheeppox vaccines that are being used more for the protection of cattle against lumpy skin disease than for the protection of sheep against sheeppox. Studies which evaluate emergency vaccination against sheeppox and post-vaccinal investigations under field conditions are very limited [7]. Therefore, the current study aimed to compare the performance of two commercially available live attenuated sheeppox vaccines for emergency vaccination against sheeppox in a highly susceptible population, which has not previously been reported in Egypt, nor worldwide, based on the best of our knowledge.

## 2. Material and Methods

### 2.1. Animals

A flock of 65 sheep in Kharsit village, with the geographical coordinates of 30°48′44″ North and 30°58′43″ East of Gharbia Governorate Egypt, was used in this study. The animals were organized into two sections—section A, 50 sheep, and section B, 15 sheep—separated by a 20 cm width mud wall of 1.5 m height and were managed by the same persons in an area of 170 m. The animals were of the Rahmani breed, of different ages and sex. They usually grazed on agricultural fields during daylight and spent their night in a fixed pen where they had concentrated rations. The Rahmani breed is usually reared for meat production, may be horned or non-horned, and has low-quality wool, which is usually of a dark color, but light colors do sometimes occur. Sheeppox had not appeared in the village for more than 30 years, and local shepherds do not vaccinate sheep against sheeppox.

### 2.2. Outbreak History

The outbreak began within section A in December 2018 after purchasing an adult ewe from outside the village. A few days after its arrival, the ewe showed systemic disease associated with variable cutaneous lesions. The animal was given Diclofenac sodium with no marked improvement and eventually died. A new case appeared within the flock, and the owner isolated it in a separate area, but the cases continued to occur. The shepherds used a topical mixture of salt and lemon on the cutaneous lesions and used Oxytetracycline ointment on the eyes. At the point of our consultation, there were 17 diseased animals and 2 deaths. A plan of action based on the treatment of the diseased cases and the emergency vaccination of the apparently healthy sheep was established. The outbreak was followed-up until it was finally contained on 28 February 2019.

### 2.3. Vaccines and Emergency Vaccination

Two commercially available sheeppox vaccines; the SPV Romanian strain attenuated on a green monkey kidney cell line and a SPV RM-65 strain attenuated on sheep kidney cells were used to compare the performance as emergency protection against sheeppox in the examined flock. Both are tissue culture-attenuated vaccines and the vaccinal dose of each contains 10^2.5^ TCID_50_, administrated subcutaneously, and stored at −20 °C for the former and at 2–8 °C for the latter. After the reconstitution of the vaccines according to the producers, 18 and 15 apparently healthy adult sheep from sections A and B were emergency vaccinated with the “SPV Romanian strain” and “SPV RM-65” vaccines, respectively. In addition, 15 animals were left unvaccinated as a control group, as this group comprised entirely of lambs and the flock owners refused to vaccinate them. Serum samples were collected from vaccinated animals to identify their non-infected status by measuring the serum protein fraction, especially α1-globulins and α2-globulins, as will be explained later in this section.

### 2.4. Sampling

Plain blood samples (each of 10 mL) were collected from the apparently healthy animals just before vaccination as well as 21 days after vaccination. Skin biopsies and plain blood were collected from the clinically-diseased animals. The biopsies were fixed in 2.5%-buffered glutaraldehyde for electron microscopy. The autopsy was performed on a dead lamb and samples from different visceral organs as well as skin lesions were collected and preserved in 10% formalin solution for histopathology. The plain blood samples were centrifuged at 5000 rpm/5 min for the separation of serum, which was preserved at −20 °C for further investigation.

### 2.5. Electron Microscopy of Skin Biopsies

The biopsies were fixed in 2.5%-buffered glutaraldehyde in 0.1 M PBS pH 7.4 at 4 °C for 2 h followed by three washing steps in PBS of 10 min each. Post-fixation in 1% osmic acid for 30 min and three similar washing steps were performed. Thereafter, the biopsies were dehydrated with an ascending series of ethyl alcohol concentrations (30, 50, 70, 90%, and absolute alcohol) for 30 min each and infiltrated with acetone for 1 h. Through transmission electron microscopy (TEM), after dehydration samples were embedded in araldite 502 resin, the plastic molds were cut in the LEICA Ultra Cut UCT ultra-microtome and stained with 1% toluidine blue. After the examination of semi-thin sections, ultra-thin sections were cut and stained with uranyl acetate, then counterstained with lead citrate and examined and photographed using JEOL-JEM-100 SX electron microscope, Japan, Electron Microscope Unit, Tanta University.

### 2.6. Serum Protein Electrophoresis

Serum protein fractions were electrophoresed by using a semiautomated agarose gel electrophoresis system (Helena Laboratories, Helena Biosciences, Sunderland, UK) according to the procedure described by the manufacturer. Five protein fractions (albumins, α1-globulins, α2-globulins, β-globulins, and γ-globulins) were identified and assessed in all serum samples. Absolute values (g/dL) for each fraction were mathematically obtained by multiplying the percentage by the total protein concentration. The albumin/globulin (A/G) ratios were calculated by dividing albumin concentrations by the sum of globulins concentrations individually.

### 2.7. Histopathology

Affected tissues were fixed in 10% neutral buffered formalin solution, and paraffin sections of five microns thick were cut by microtome, stained with hematoxylin and eosin, and examined under a light microscope, as previously described [12].

### 2.8. Statistical Analysis

Statistical analysis was performed using SPSS (IBM SPSS Statistics for Windows, Version 22.0. IBM Corp., Armonk, NY, USA). The incidence of sheeppox among different groups of the flock, including 17 non-vaccinated adult animals (diseased adults before interference), 15 non-diseased lambs as (controls after vaccination), vaccinated sheep with SPV Romanian strain (*n* = 18), and vaccinated sheep with SPV RM-65 (*n* = 15), was calculated by the evaluation of the cumulative incidence (CI) of infection and the case fatality (CF) rate among different groups of animals as described by Dicker et al., 2006 [13], using the following formulas:

$$CI=\frac{Number\;of\;new\;cases\;of\;disease\;during\;specified\;period}{Size\;of\;population\;at\;risk\;at\;start\;of\;period}\times 100\;CF=\frac{Number\;of\;sheep\;pox\;cases\;which\;die}{number\;of\;sheep\;pox\;cases}\times 100$$

Chi square and Fisher’s exact tests were used to examining the statistical difference between the cumulative incidence of sheeppox and case fatality of sheeppox among different groups at a significant level of difference of *p* < 0.05.

## 3. Results

**Figure 1:**
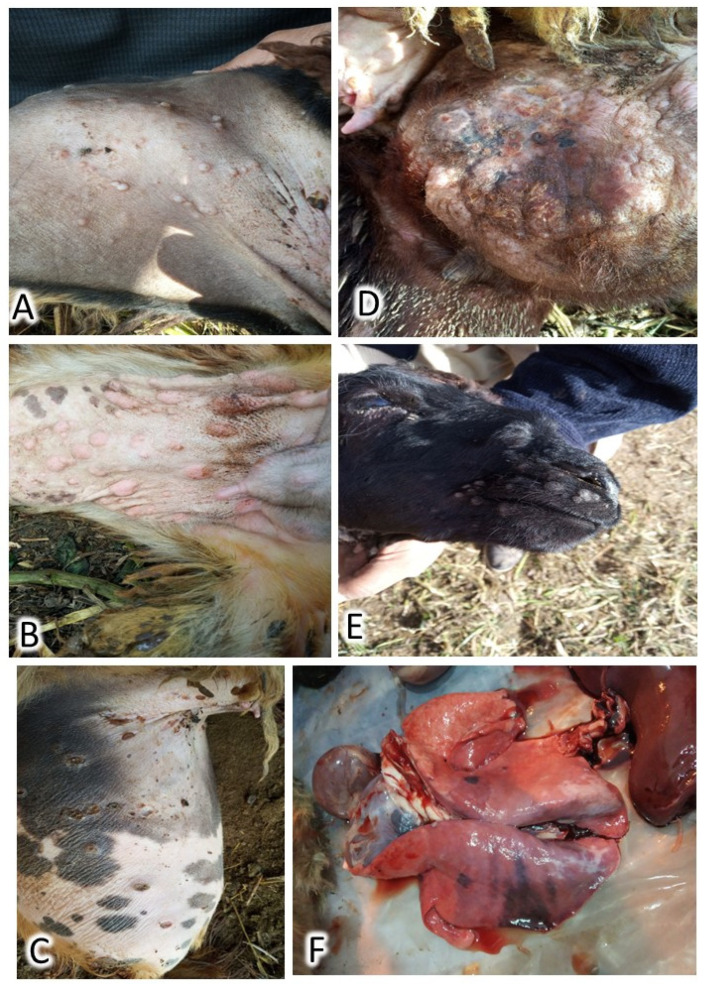
Gross pathology of sheeppox lesions: papular sheeppox lesions spread over the inner aspect of the fatty tail (**A**), variable size papules in the inner thigh region (**B**), dry thick scab lesions over the inner aspect of the fatty tail (**C**), abnormal udder showing apparent gross sheeppox lesions (**D**), variable size papules on the upper and lower lips (**E**), and black patch spots over the lung of a deceased sheep (**F**).

During the first visit to the herd, seventeen (26.1%) animals were diseased, of which two (3.1%) adults had died. Cases showed cutaneous lesions in the form of small, numerous lesions that begin with macule and end with scar formation. These lesions were extremely visible in wool-less areas of the body: the ventral abdomen, the inner aspect of the fatty tail, the perineal region, genitalia, axilla, groin, and head, as illustrated in Figure 1. Pyrexia, enlarged lymph nodes, nasal and ocular discharges, palpebral edema, poor appetite, hurried respiration, and exaggerated vesicular lung sound were detected. Postmortem examination revealed a marked congestion of all visceral organs and notable pulmonary lesions (Figure 1). The outbreak ended 2.5 months after the appearance of the first case. At the end of the outbreak, a total of 43 sheep had contracted the disease, of which 22 cases died, indicating a cumulative incidence of 66.2% and an overall case fatality of 51.1% (Table 1).

There were no recorded cases of sheeppox infection or mortalities among the SPV RM-65-vaccinated animals of section B, and this results in significant low cumulative incidence and case fatality among this group of animals compared to the other groups at *p* < 0.05 (Table 1)—only slight depression and laziness were observed in a few cases as a post-vaccinal reaction. On the other hand, there was no significant difference in incidence among other groups: non-vaccinated adults, non-vaccinated lambs, and SPV Romanian strain vaccinated adult animals; however, the clinical signs were milder among the last group. The case fatality was not significantly different between non-vaccinated adults and those SPV Romanian strain-vaccinated adult animals (Table 1), while the case fatality among non-vaccinated lambs was significantly higher than all other groups at *p* < 0.05 (Table 1).

**Figure 2:**
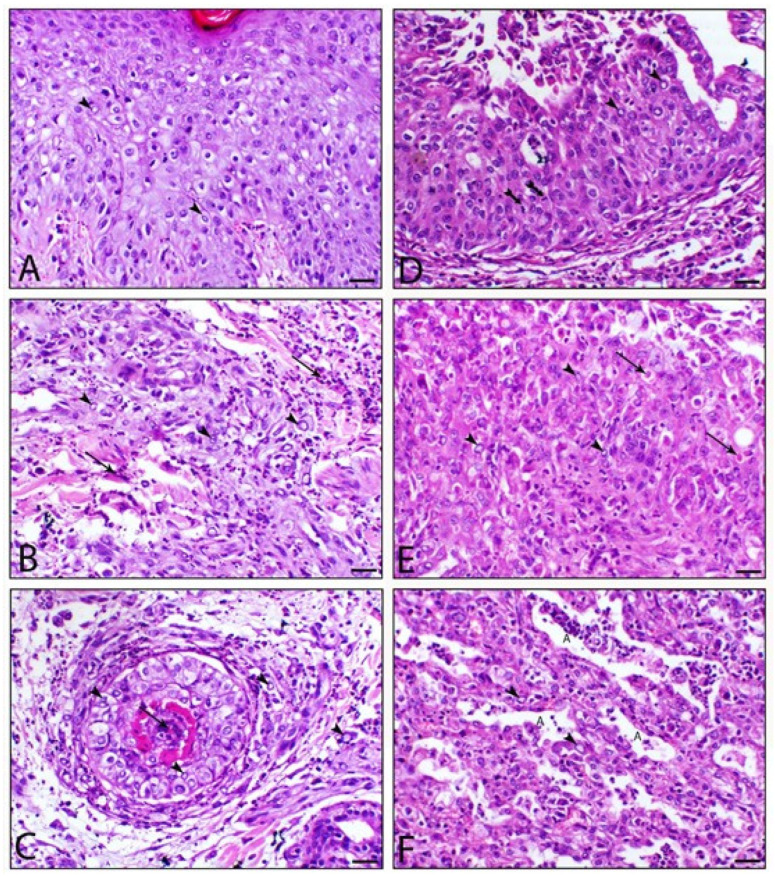
Cutaneous and pulmonary sections of infected sheep with SPV. Figures (**A**–**C**) represent the skin, and Figures (D–**F**) represent the lung. Epidermal layer showing ballooning degeneration with macrovesicles formation associated with the presence of intra-epithelial sheeppox cells (arrowheads), (**B**) dermis showing dermatitis associated with the necrosis of the collagen with marked inflammatory cell infiltration of mostly macrophages and histocytes and sheeppox cells (arrowhead) and (**C**) showing the necrosis of hair complex with marked infiltration of sheeppox cells. (**D**,**E**) Bronchial section showing the stratification of the lining epithelium associated with the degeneration of epithelial cells, the hyperplasia of the basal epithelial cells (tailed-arrow), and intracytoplasmic inclusion bodies (arrows). (**F**) Feature of interstitial pneumonia associated with marked thickening of the interalveolar septa and the marked infiltration of macrophages, histocytes, and sheeppox cells (arrowhead), A letter indicates atelectatic alveolar spaces, H&E, bar = 50 µm.

**Figure 3:**
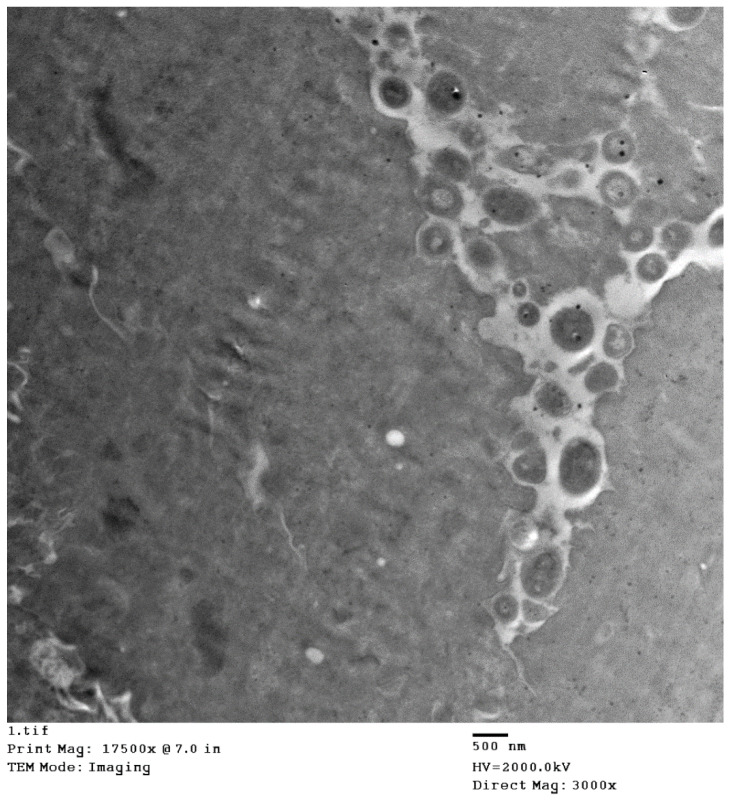
Electron microscopy of skin showing intra-cytoplasmic brick-shaped sheeppox virions.

The histopathology of the skin, as well as organs, indicated the existence of sheeppox cells in cutaneous and pulmonary tissues and intracytoplasmic inclusion bodies were also detected. Detailed histopathological findings are illustrated in Figure 2. The electron microscopy of skin biopsies revealed intracytoplasmic brick-shaped sheeppox virions of about 500 nm in size (Figure 3).

The serum protein fractionation of the healthy and diseased animals as well as for the two vaccinated groups is illustrated in Table 2. There were no significant differences between mean values of serum total protein concentrations between all animal groups (Table 2; *p* > 0.05). Serum proteins were separated by using agarose gel electrophoresis and five fractions (albumin, α1, α2, β, and γ) were identified in all serum samples. The absolute values of albumin were decreased in diseased animals and significantly lower than those of healthy animals (Table 2; *p* < 0.05). In contrast, the absolute values of α1- and α2-globulins were significantly increased in diseased compared to heathy animals (Table 2; *p* < 0.05). Furthermore, there was a significant decrease in the absolute values of albumin and a significant increase in the absolute values of β-globulins in the Romanian strain-immunized group compared to healthy animals (Table 2; *p* < 0.05). However, the absolute values of α1- and β-globulins were significantly higher in the RM-65-immunized group when compared to the healthy animals (Table 2; *p* < 0.05). Moreover, there were no significant differences between the mean values of γ-globulins between all groups (Table 2; *p* > 0.05). Lastly, infected animals, the Romanian strain-immunized group and the RM-65-immunized group had lower A/G ratios compared to healthy animals (Table 2; *p* < 0.05)

## 4. Discussion

The control of sheeppox depends mainly on biosecurity practices and immunization using live attenuated vaccines [1], although these measures are usually performed in organized sheep farms, not in small pastoral flocks as reported in the current study. The insidious nature of sheeppox necessitates the use of sheeppox vaccines, otherwise the disease may enter the sheep population invisibly, similarly to the scenario of this outbreak. All of the currently available vaccinations are manufactured using primary cells; therefore, they may be accompanied by quality concerns as well as other issues. The Capripox vaccines may have protection-related problems that call for the upgrading of the control strategies against these illnesses [4]. Therefore, quality control for sheeppox vaccine production is required [9].

Despite the fact that emergency vaccination has been widely reported in cattle against lumpy skin disease [8,10], emergency vaccination in the presence of sheeppox cases was previously mentioned only in a limited and indirect manner in the scientific literature. To the best of our knowledge, the current study may be the first to test the reliability of emergency vaccination against sheeppox in a clear, direct way. The obtained results indicated that the RM-65 vaccine demonstrated a better performance than the Romanian strain vaccine for emergency protection against sheeppox in Egypt, as no cases appeared after its use. However, the occurrence of outbreaks among locally produced RM-65-vaccinated sheep in Algeria has been reported [9].

The Romanian vaccine has been demonstrated to be effective to protect sheep and goats against sheeppox and goatpox [14]. The manufacturing and quality control of sheeppox vaccines has been thoroughly explained in the OIE terrestrial manual (2017) [6], starting with the characteristics of the seed strain and continuing through the production and testing of the final product [6]. This is crucial to take into account the manufacturing of sheeppox vaccines, not only as general guidelines but also at a country or regional level with ongoing updates to the vaccine based on the epidemiological condition and the circulating strains.

The analysis of serum proteins and their electrophoretic separations have been studied intensively and constitute a vital component of laboratory diagnostic evaluations in small animal medicine, especially in support of a clinical diagnosis of diseases characterized by dysproteinemia or to identify the presence of inflammation with increased α-globulins [15].

The data of the present study revealed a significant decrease in the absolute values of albumin in the infected animals when compared to the healthy animals. The decrease in the concentrations of albumin is a common form of dysproteinemia. Basically, the decrease can be attributed to either albumin loss or failure of albumin synthesis. Serum albumin is the major negative acute-phase protein (APP), and its synthesis may be markedly reduced during the acute phase response (APR). The APR is part of the early-defense or innate immune system, which is triggered by different stimuli, including trauma, infection, and inflammation. The synthesis of positive acute-phase proteins is markedly increased during the acute inflammatory processes. These reactions require a great number of amino acids. Thus, albumin synthesis is downregulated, and amino acids are used mainly for the synthesis of the positive acute-phase proteins [16].

Additionally, our data revealed a significant increase in the absolute concentration of α1- and α2-globulins in infected animals. As many of the APPs migrate to the α1- and α2-globulin regions, increases in these globulins are a common finding in acute inflammatory diseases and are caused by the activation of the host inflammatory responses [17,18].

The data of this study also showed a significant increase in the absolute values of β-globulins in the Romanian strain- and RM-65-immunized groups only in comparison to the control group. Important acute-phase proteins, such as transferrin, β_2_-microglobulin, and C-reactive protein, migrate into the β-region fraction [19]. So, several inflammatory diseases and infections may also be accompanied by increases in the β-fraction as a result of the elevated production of these proteins. Moreover, one of the main components of β-region fraction increases in response to the antigenic stimulation. Furthermore, in response to antigenic stimulation, the cells of the adaptive immune system produce different immunoglobulins (Igs). Some of these Igs, such as IgM, IgA, and IgE, may migrate into the β-γ zone or β-region [20].

Finally, infected, Romanian strain- and RM-65-immunized groups had lower A/G ratios compared to the healthy group before vaccination. Decreased A/G ratio may be associated with the overproduction of globulins, the decreased synthesis of albumin, or the loss of albumin from circulation [21]. Basically, alterations in the absolute values of albumin and globulins fractions lead to changes in the albumin:globulin (A/G) ratio. Thus, the interpretation of the A/G ratio is very important in providing information about the changes in the pattern of serum proteins and could help in the classification and identification of dysproteinemias [22]. New antigenic components for the diagnosis of and protection against sheeppox using advanced recombinant technology [23] are still required to achieve more progress in combating this disease.

## 5. Conclusions

In conclusion, the findings of this study indicate that the SPV RM-65 vaccine demonstrated better performance for emergency vaccination under field conditions in Egypt against sheeppox in comparison to the Romanian strain vaccine. However, wider investigatory studies are still required, not only for the evaluation of the emergency use of sheeppox vaccines but also to investigate the protective potentials of these vaccines in a comparative way, considering vaccine production issues and their effect on vaccine efficacy.

## Figures and Tables

**Table 1:** The incidence and case fatality of sheeppox among different groups of sheep.

Groups	Total	Diseased (%)	Case Fatality (%)
Non-vaccinated healthy Lambs	15	12 (80%) ^a^	12 (100%) ^b^
Non-vaccinated diseased Adults	17	17 (100%) ^a^	5 (29.4%) ^a^
SPV Romanian strain-vaccinated adult animals	18	14 (78%) ^a^	5 (35.7%) ^a^
SPV RM-65-vaccinated adult animals	15	0 (0%) ^b^	0 (0.0%)
Total	65	43 (66.2%)	22 (51.1%)

In the same column, the categories with different small letters are significantly different at *p* < 0.05.

**Table 2:** Protein fractionation of the healthy, diseased, and vaccine A- and B-immunized animals.

Animals	Parameters						
	TP g/L	Albumin g/L	α1-Globulin g/L	α2-Globulin g/L	β-Globulin g/L	γ-Globulin g/L	A/G Ratio
Healthy before vaccination	6.53 ± 0.18 ^a^	3.20 ± 0.06 ^a^	0.10 ± 0.02 ^c^	0.38 ± 0.05 ^b^	0.75 ± 0.04 ^b^	2.10 ± 0.18 ^a^	0.97 ± 0.09 ^a^
Diseased	6.83 ± 0.29 ^a^	2.94 ± 0.09 ^b^	0.31 ± 0.07 ^a^	0.77 ± 0.10 ^a^	0.89 ± 0.04 ^ab^	1.90 ± 0.12 ^a^	0.76 ± 0.05 ^b^
Romanian strain-vaccinated	6.20 ± 0.60 ^a^	2.67 ± 0.17 ^c^	0.14 ± 0.02 ^bc^	0.42 ± 0.10 ^b^	0.98 ± 0.19 ^a^	2.00 ± 0.13 ^a^	0.76 ± 0.05 ^b^
RM-65-vaccinated	6.65 ± 0.15 ^a^	3.05 ± 0.04 ^ab^	0.18 ± 0.00 ^b^	0.48 ± 0.03 ^b^	0.96 ± 0.05 ^a^	2.00 ± 0.04 ^a^	0.85 ± 0.02 ^b^

Data are expressed as mean ± SD. Mean values in the same row bearing different superscript letters were significantly differ at *p* < 0.05.

## Data Availability

All the data are presented in this study.
